# Supplementary material for: Impact of a UV-C Scalable Reactor on the Chemical and Sensory Quality of Peppercorns
Source: Foods. 2025 Aug 29;14(17):3056. doi: 10.3390/foods14173056 (PMC12428542; doi:10.3390/foods14173056)
Supplement: Supplementary file 1 [file foods-14-03056-s001.zip › foods-3824577-supplementary.pdf]

Table S1. Standardized area per gram of sample before and after the UV treatment for every analyzed volatile. Results are expressed as mean ± SD (n=3).

Sample	Treatment	Beta-pinene	3-Carene	D-limonene	Alpha-copaene	Caryophyllene	Beta-bisabolene	Beta-phellandrene
Black pepper	Control	0.162 ± 0.004 [a]	0.047 ± 0.002 [a]	1.08 ± 0.03 [a]	2.3 ± 0.1 [a]	6.7 ± 0.3 [a]	1.10 ± 0.03 [a]	0.253 ± 0.006 [a]
	Agitation	0.21 ± 0.07 [ab]	0.045 ± 0.002 [a]	1.26 ± 0.21 [a]	2.6 ± 0.4 [a]	6.9 ± 0.3 [a]	1.4 ± 0.3 [a]	0.18 ± 0.06 [a]
	UV	0.147 ± 0.002 [b]	0.044 ± 0.001 [a]	0.93 ± 0.01 [b]	2.06 ± 0.04 [b]	6.2 ± 0.1 [b]	0.97 ± 0.05 [b]	0.247 ± 0.006 [a]
White pepper	Control	0.18 ± 0.02 [a]	0.97 ± 0.05 [a]	0.53 ± 0.02 [a]	0.006 ± 0.002 [a]	2.89 ± 0.07 [a]	0.074 ± 0.003 [a]	ND
	Agitation	0.15 ± 0.05 [a]	0.89 ± 0.06 [a]	0.45 ± 0.05 [a]	0.007 ± 0.002 [a]	2.43 ± 0.31 [a]	0.070 ± 0.003 [a]	ND
	UV	0.062 ± 0.003 [b]	0.43 ± 0.02 [b]	0.305 ± 0.004 [b]	0.005 ± 0.003 [a]	2.77 ± 0.09 [a]	0.074 ± 0.005 [a]	ND
Green pepper	Control	0.33 ± 0.02 [a]	0.76 ± 0.06 [a]	0.55 ± 0.04 [a]	0.026 ± 0.001 [a]	1.57 ± 0.08 [a]	0.224 ± 0.005 [a]	0.099 ± 0.007 [a]
	Agitation	0.34 ± 0.04 [a]	0.73 ± 0.04 [a]	0.52 ± 0.03 [a]	0.022 ± 0.05 [ab]	1.40 ± 0.11 [a]	0.218 ± 0.004 [a]	0.095 ± 0.005 [a]
	UV	0.28 ± 0.04 [a]	0.69 ± 0.08 [a]	0.48 ± 0.04 [a]	0.0193 ± 0.0007 [b]	1.64 ± 0.02 [a]	0.21 ± 0.01 [a]	0.08 ± 0.006 [b]
Pink pepper	Control	0.23 ± 0.02 [a]	2.1 ± 0.2 [a]	2.3 ± 0.2 [a]	0.13 ± 0.02 [a]	1.5 ± 0.2 [a]	ND	0.57 ± 0.06 [a]
	Agitation	0.19 ± 0.02 [a]	1.9 ± 0.3 [a]	2.4 ± 0.7 [a]	0.13 ± 0.01 [a]	1.4 ± 0.1 [a]	ND	0.56 ± 0.12 [a]
	UV	0.061 ± 0.001 [b]	0.262 ± 0.004 [b]	0.573 ± 0.007 [b]	0.121 ± 0.004 [a]	1.55 ± 0.03 [a]	ND	0.11 ± 0.02 [b]

Notes: Different letters for a compound within the same sample indicate significant differences ($p<0.05$) between mean values.

Table S1. (Continued)

Sample	Treatment	o-Cymene	Caryophyllene oxide	Beta-myrcene	Linalool	Alpha-phellandrene	Sabinene	Germacrene-D
Black pepper	Control	ND	0.25 ± 0.04 [a]	0.0427 ± 0.0009 [a]	0.0082 ± 0.0002 [a]	0.017 ± 0.001 [a]	0.238 ± 0.006 [a]	0.206 ± 0.009 [a]
	Agitation	ND	0.32 ± 0.08 [a]	0.048 ± 0.003 [a]	0.008 ± 0.001 [a]	0.011 ± 0.005 [ab]	0.18 ± 0.05 [a]	0.27 ± 0.05 [a]
	UV	ND	0.3 ± 0.08 [a]	0.0378 ± 0.0005 [b]	0.0078 ± 0.0003 [a]	0.0121 ± 0.0002 [b]	0.252 ± 0.004 [b]	0.178 ± 0.006 [b]
White pepper	Control	0.161 ± 0.009 [a]	0.25 ± 0.04 [a]	0.051 ± 0.003 [a]	0.042 ± 0.001 [a]	0.078 ± 0.004 [a]	ND	ND
	Agitation	0.14 ± 0.3 [a]	0.20 ± 0.05 [a]	0.041 ± 0.012 [a]	0.034 ± 0.007 [ab]	0.064 ± 0.006 [b]	ND	ND
	UV	0.084 ± 0.003 [b]	0.27 ± 0.02 [a]	0.0183 ± 0.0007 [b]	0.0367 ± 0.0003 [b]	0.0321 ± 0.0007 [c]	ND	ND
Green pepper	Control	0.053 ± 0.003 [a]	0.056 ± 0.008 [a]	0.053 ± 0.005 [a]	0.025 ± 0.002 [a]	0.019 ± 0.001 [a]	0.39 ± 0.03 [a]	ND
	Agitation	0.050 ± 0.003 [a]	0.052 ± 0.007 [a]	0.048 ± 0.006 [a]	0.020 ± 0.004 [a]	0.012 ± 0.006 [a]	0.41 ± 0.04 [a]	ND
	UV	0.051 ± 0.003 [a]	0.05 ± 0.02 [a]	0.047 ± 0.008 [a]	0.028 ± 0.001 [a]	0.019 ± 0.006 [a]	0.28 ± 0.04 [b]	ND
Pink pepper	Control	0.57 ± 0.07 [a]	0.057 ± 0.007 [a]	0.72 ± 0.08 [a]	ND	2.4 ± 0.3 [a]	0.18 ± 0.02 [a]	ND
	Agitation	0.57 ± 0.04 [a]	0.050 ± 0.008 [a]	0.60 ± 0.06 [ab]	ND	2.3 ± 0.2 [a]	0.18 ± 0.04 [a]	ND
	UV	0.157 ± 0.009 [b]	0.067 ± 0.004 [a]	0.0538 ± 0.0009 [b]	ND	0.23 ± 0.02 [b]	0.0466 ± 0.0006 [b]	ND

Notes: Different letters for a compound within the same sample indicate significant differences ($p<0.05$) between mean values.

Table S1. (Continued)

Sample	Treatment	Beta-elemene	Humulene	Delta-cadiene	Alpha-pinene	Alpha-terpinolene
Black pepper	Control	0.122 ± 0.003 [a]	0.46 ± 0.02 [a]	0.453 ± 0.008 [a]	0.0318 ± 0.0008 [a]	0.0106 ± 0.0009 [a]
	Agitation	0.122 ± 0.005 [a]	0.49 ± 0.05 [a]	0.475 ± 0.009 [a]	0.0322 ± 0.0012 [a]	0.0068 ± 0.0001 [b]
	UV	0.111 ± 0.004 [b]	0.41 ± 0.02 [b]	0.385 ± 0.008 [b]	0.0334 ± 0.0007 [a]	0.0070 ± 0.0001 [b]
White pepper	Control	0.078 ± 0.003 [a]	0.169 ± 0.005 [a]	ND	0.062 ± 0.006 [a]	0.0203 ± 0.0001 [a]
	Agitation	0.061 ± 0.006 [a]	0.137 ± 0.012 [a]	ND	0.052 ± 0.005 [a]	0.017 ± 0.004 [a]
	UV	0.078 ± 0.003 [a]	0.168 ± 0.007 [a]	ND	0.023 ± 0.001 [b]	0.0115 ± 0.0003 [b]
Green pepper	Control	0.095 ± 0.004 [a]	0.098 ± 0.002 [a]	ND	0.168 ± 0.001 [a]	0.0049 ± 0.0002 [a]
	Agitation	0.086 ± 0.008 [a]	0.089 ± 0.009 [a]	ND	0.176 ± 0.006 [a]	0.0038 ± 0.0009 [a]
	UV	0.097 ± 0.002 [a]	0.100 ± 0.003 [a]	ND	0.13 ± 0.02 [b]	0.005 ± 0.001 [a]
Pink pepper	Control	0.31 ± 0.05 [a]	0.21 ± 0.04 [a]	0.28 ± 0.005 [a]	0.70 ± 0.04 [a]	0.36 ± 0.04 [a]
	Agitation	0.28 ± 0.04 [a]	0.20 ± 0.06 [a]	0.24 ± 0.006 [a]	0.67 ± 0.05 [a]	0.34 ± 0.06 [a]
	UV	0.339 ± 0.006 [a]	0.265 ± 0.004 [a]	0.363 ± 0.005 [b]	0.329 ± 0.004 [b]	0.13 ± 0.005 [b]

Notes: Different letters for a compound within the same sample indicate significant differences ($p<0.05$) between mean values.

Table S2. Quantitative analysis of phenolic compounds and organic acids in UV-treated and untreated peppercorn samples. Results are expressed in mg/kg as mean ± SD.

Compound	Sample	Black pepper		Green pepper		White pepper		Pink pepper	
	Treatment time (min)	0	90	0	90	0	90	0	90
Quinic acid		9.2 ± 0.7	9.0 ± 2.2	1.9 ± 0.3	2.6 ± 0.3*	3.7 ± 0.6	5.1 ± 0.4*	8.1 ± 0.4	7.1 ± 0.6
Succinic acid		248 ± 23	304 ± 41	65 ± 3	63 ± 2	46 ± 3	46 ± 2	82 ± 6	92 ± 8
Gallic acid		0.44 ± 0.09	1.18 ± 0.30*	2.46 ± 0.63	0.46 ± 0.03*	0.36 ± 0.02	0.49 ± 0.03*	685 ± 46	606 ± 46
Protocatechuic acid		21.8 ± 1.5	20.7 ± 2.0	7.2 ± 0.4	7.9 ± 0.3	7.4 ± 0.4	7.8 ± 0.7*	45.3 ± 1.8	39.6 ± 1.8*
Catechin		1.2 ± 0.5	1.4 ± 0.3	29.3 ± 1.0	29.8 ± 2.7	6.3 ± 0.6	7.0 ± 0.1	350 ± 50	284 ± 30
p-Hydroxibenzoic acid		6.8 ± 0.5	6.7 ± 0.7	1.6 ± 0.2	1.6 ± 0.1	4.7 ± 0.2	4.6 ± 0.1	5.6 ± 0.2	5.5 ± 0.3
Vanillic acid		9.0 ± 0.4	7.2 ± 1.1	3.3 ± 1.1	0.6 ± 0.2*	5.3 ± 1.8	8.8 ± 0.8*	ND	ND
Syringic acid		19 ± 2	18 ± 1	12.4 ± 0.7	11.1 ± 0.8	8.8 ± 1.9	11.7 ± 1.6	0.41 ± 0.05	0.32 ± 0.01
Epicatechin		0.29 ± 0.04	0.29 ± 0.05	8.6 ± 0.5	8.8 ± 0.2	0.46 ± 0.03	0.53 ± 0.07	0.63 ± 0.13	0.53 ± 0.06
Caffeic acid		17.5 ± 2.4	4.4 ± 1.2*	2.2 ± 0.2	1.9 ± 1.0	3.0 ± 0.6	2.8 ± 0.8	9.5 ± 1.4	16.2 ± 3.1*
Vanillin		76 ± 5	87 ± 14	82 ± 4	85 ± 3*	86 ± 5	75 ± 3*	37 ± 9	38 ± 5
Coumaric acid		0.48 ± 0.01	0.35 ± 0.07*	0.09 ± 0.01	0.12 ± 0.03	0.14 ± 0.03	0.12 ± 0.04	3.69 ± 0.07	4.14 ± 0.12*
Ferulic acid		3.6 ± 0.2	3.5 ± 0.5	3.3 ± 0.4	3.0 ± 0.5	1.6 ± 0.1	1.5 ± 0.1	1.3 ± 0.1	1.6 ± 0.2
Quercetin		ND	ND	ND	ND	ND	ND	5.8 ± 2.1	6.1 ± 1.4
Cinnamic acid		28 ± 15	25 ± 13	34 ± 1	26 ± 10	32 ± 19	42 ± 15	12 ± 6	25 ± 6*

Notes: An asterisk indicates significant differences ($p<0.05$) between mean values (treated and untreated) for a that compound and sample.

Table S3. Qualitative analysis of phenolic compounds and organic acids in UV-treated and untreated peppercorn samples. Results are expressed in standardized signal per gram as mean ± SD.

Compound	Sample	Black pepper		Green pepper		White pepper		Pink pepper	
	Treatment time (min)	0	90	0	90	0	90	0	90
**Neochlorogenic acid**		$9x10^3$ ± 1 $x10^3$	8 $x10^3$ ± 1 $x10^3$	8 $x10^3$ ± 2 $x10^3$	16 $x10^3$ ± 2 $x10^3$*	7 $x10^3$ ± 2 $x10^3$	8 $x10^3$ ± 1 $x10^3$	37 $x10^3$ ± 7 $x10^3$	27 $x10^3$ ± 4 $x10^3$
**Chlorogenic acid**		ND	ND	ND	ND	ND	ND	16 $x10^3$ ± 3 $x10^3$	12 $x10^3$ ± 3 $x10^3$
**Rutin**		ND	ND	ND	ND	ND	ND	ND	ND
**Phlorizin**		ND	ND	ND	ND	ND	ND	13 $x10^3$ ± 1 $x10^3$	12 $x10^3$ ± 1 $x10^3$
**Kaempferol**		ND	ND	ND	ND	ND	ND	109 $x10^3$ ± 2 $x10^3$	130 $x10^3$ ± 30 $x10^3$
**Hesperidin**		ND	ND	ND	ND	ND	ND	ND	ND

Notes: An asterisk indicates significant differences ($p<0.05$) between mean values (treated and untreated) for a that compound and sample.
